# Tension band plating of an anterior tibial stress fracture nonunion in an elite athlete, initially treated with intramedullary nailing: a case report

**DOI:** 10.1186/s13256-018-1718-8

**Published:** 2018-06-29

**Authors:** George A. Tsakotos, Anastasios V. Tokis, Christos G. Paganias

**Affiliations:** 1 Department of Orthopedics and Sports Medicine, Metropolitan Hospital, Athens, Greece; 2 Department of Orthopedics, Doctors’ Hospital, Athens, Greece

**Keywords:** Stress fractures, Tibial stress fractures, Anterior tibial, Tension band plate

## Abstract

**Background:**

Leg pain in athletes is a common condition and is often related to tibial stress fracture. When non-operative treatment fails, the optimal surgical treatment is controversial. The aim of this study was to report a case of tension band plating of an anterior tibial stress fracture nonunion, treated previously with intramedullary nailing. To the best of our knowledge, this is the first reported case in which tension band plating was placed without removing the preexisting intramedullary nail.

**Case presentation:**

The tibial shaft is a common location of stress fracture in athletes. Anterior tibial stress fractures are difficult to manage. When conservative treatment fails, intramedullary nailing is the mainstay of treatment. However, nonunion is a serious complication. In our case, a non-united anterior tibial stress fracture, treated with intramedullary nailing, was addressed with the application of a compression prebended plate over the nail in a 23-year-old French man of African origin who is an elite football player. At 3-months postoperatively he was pain free and started light exercises. At 6-months postoperatively, complete radiologic union of the fracture was evident. He was symptom free; he resumed at that time a full training program and he returned to play football at preinjury high competition level.

**Conclusions:**

Compression plating is a valuable method of treating non-united anterior tibial stress fractures. We believe that anterior tension band plating is superior to intramedullary nailing in managing anterior tibial stress fractures, not only after failure of intramedullary nailing, but also as a first-line surgical treatment. This technique offers advantages, such as no violation of the extensor mechanism and risk of anterior knee pain, and directly addresses the underlying problem of distraction forces acting on the anterior tibial cortex and compromising fracture healing. Especially in high-level athletes, who cannot tolerate prolonged inactivity, early surgical intervention of anterior tibial stress fractures with tension band plating is a reliable option that can accelerate recovery.

## Background

Leg pain in athletes is a very common condition. This may be due to medial tibial stress syndrome, chronic exertional compartment syndrome, nerve entrapment, popliteal artery entrapment, deep vein thrombosis, and stress fracture [1, 2].

Stress fractures were first described in 1855 [3]. They mostly occur in athletes, military recruits, and patients with endocrine or nutritional abnormalities. In elite football players, stress fractures may have an injury incidence of 0.04 injuries/1000 hours and a football team can expect one stress fracture every third season [4]. Stress fractures are rare in professional football players but cause long absences from the game.

Stress fracture is the result of an imbalance between bone formation and bone reabsorption during excessive, repetitive, dynamic cyclic loading. Multiple factors contribute to this condition including age, sex, race, nutritional behaviors, hormonal status, medication and tobacco use, bone structure, bone density, muscle length, muscle strength and tightness, joint range of motion, training volume, type of activity, training surface, shoes, and environmental conditions [5, 6]. Younger age and intensive pre-season training are important risk factors. Osteoclastic activity occurs faster than osteoblastic activity and bone mineral reabsorption exceeds deposition, so a fracture occurs.

The tibial shaft is a common location of stress fractures in athletes. Tibial stress fractures are divided into two groups: anterior and posterior-posteromedial [7]. Posterior-posteromedial cortex fractures are more frequent and respond well to conservative treatment. Anterior tibial cortex fractures are less common, approximately 2.7 to 4.6% of stress fractures [8]. This injury is derived from excessive tensile forces from posterior muscle activity. The anterior tibial cortex is a poorly vascularized area without sufficient muscle and soft tissue coverage. When repetitive forces are applied to this “watershed” area, the bone may not be able to heal and, moreover, this predisposes the fracture to being refractory to conservative treatment and progress to complete fracture, delayed union, or nonunion [9–11].

Conservative treatment is the mainstay of treatment for the majority of tibial stress fractures, including rest, activity modification, use of an orthosis, ultrasound stimulation, and electromagnetic field therapy. When the conservative treatment of tibial stress fractures fails, a surgical operation is recommended. Intramedullary (im) nailing is the most common modality of treatment.

We present a case where an anterior tibial stress fracture in an elite athlete did not heal after im nailing. To address this nonunion, tension band plating with bone grafting was performed. In order to avoid further damage to the extensor mechanism of the knee, the im nail was not removed. With this technique, fracture union was achieved and the athlete returned to play football at a high level at 6-months postoperatively. To the best of our knowledge, this is the first reported case of a tibial stress fracture nonunion that was treated with tension band plating and without removing the preexisting im nail.

## Case presentation

A 23-year-old French man of African origin, an elite football player, sustained a midshaft anterior cortex tibial stress fracture 2.5 years ago. Initially, he was treated with cast immobilization, no weight bearing for 3 months, ultrasound stimulation, and electromagnetic field therapy. The fracture did not heal; he had pain during gait, so he continued no weight bearing for 3 additional months. After that period, the fracture site still was not healed, so he underwent an operation performed by his team doctor. In this operation, the medullary canal of his tibia was reamed and an im nail was inserted.

**Fig. 1:**
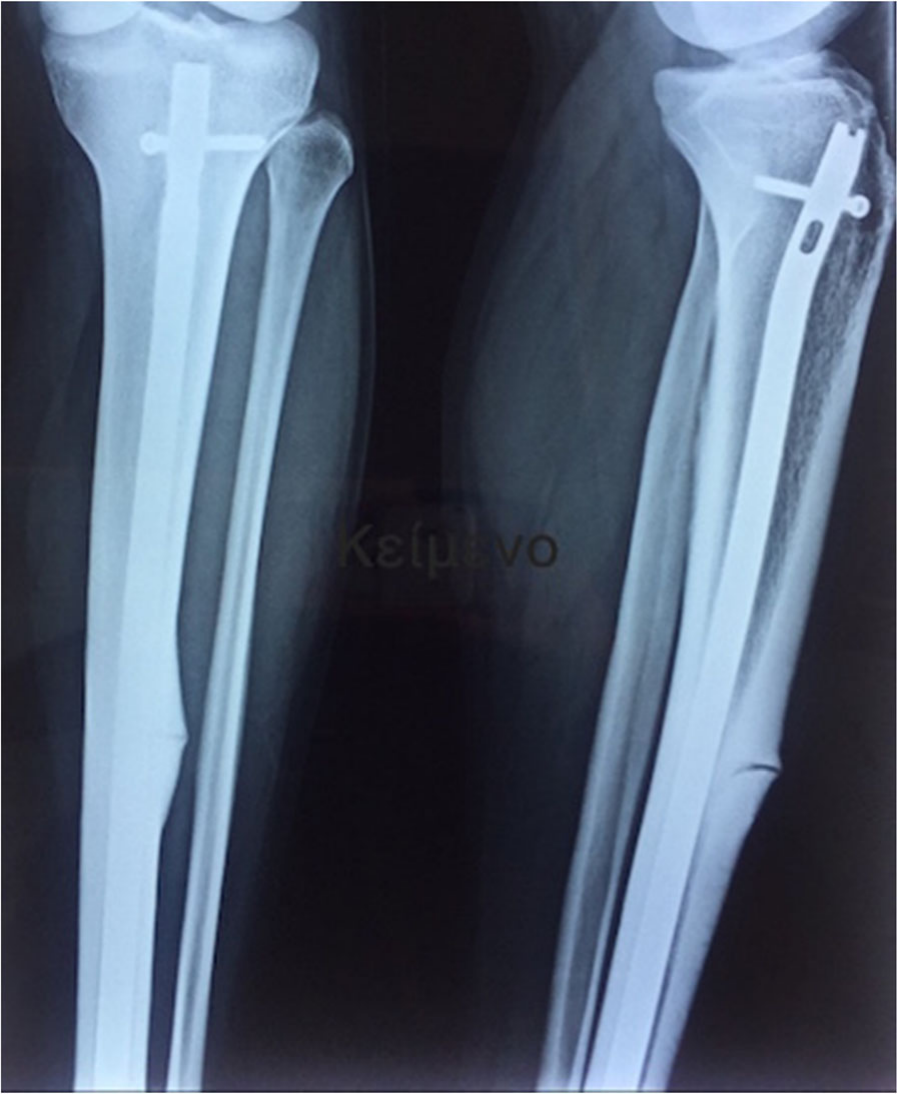
Anteroposterior and lateral X-ray of the tibia, 18 months after intramedullary nailing. The presence of a nonunion is obvious

Unfortunately, the fracture site did not consolidate again, even 18-months postoperatively, so he presented to our clinic for counseling. It was obvious from the X-ray (Fig. 1) that a nonunion of the fracture had occurred.

He did not smoke tobacco and he had a free medical history. When he presented to our clinic, the area at the fracture site was swollen and painful when palpated. The pain got worse when he attempted to walk with full weight bearing, so he had to use crutches. An examination of the peripheral nervous system of his lower extremities did not provide us with any pathologic findings. In addition, the laboratory examinations for possible endocrine or metabolic disorders were negative (Table 1), so he was advised to have a reoperation to address this nonunion. The treatment options for such cases include nail exchange, drilling of the fracture site, bone grafting, or removal of the nail and internal fixation with a plate. We performed a tension band plate fixation, which is a technique already described for the treatment of anterior tibial stress fractures that failed non-operative treatment [12], with bone grafting and without removing the nail.

**Table 1:** Patient’s laboratory examination results

Examination	Patient’s value	Normal range
RBC	5,620,000/μL	4,500,000–5,700,000/μL
Hgb	15.6 g/dL	13.5–17.5 g/dL
Hct	46.9%	42–54%
WBC	6910/μL	4100–11000/μL
Neutrophils	59%	40–75%
Lymphocytes	29.3%	20–40%
Mononuclear	8.4%	2–10%
Eosinophils	2.7%	1–6%
Basophils	0.6%	0–1%
Platelets	259,000/μL	150,000–400,000/μL
ESR (first hour)	6 mm	0–10 mm
Glucose	82 mg/dL	70 –110 mg/dL
Creatinine	1.03 mg/dL	0.7–1.5 mg/dL
AST (SGOT)	17 U/L	< 40 U/L
ALT (SGPT)	18 U/L	< 40 U/L
ALP	188 U/L	64–306 U/L
TSH	2.46 μIU/mL	0.35–4.94 μIU/mL
Calcium	9.8 mg/dL	8.2–10.6 mg/dL
Albumin	4.6 g/dL	3.5–5.0 g/dL
25-OH vitamin D	38 ng/mL	30–100 ng/mL
iPTH	33 pg/mL	10–65 pg/mL

*25-OH vitamin D* calcifediol, *ALP* alkaline phosphatase, *ALT* alanine aminotransferase, *AST* aspartate aminotransferase, *ESR* erythrocyte sedimentation rate, *Hct* hematocrit, *Hgb* hemoglobin, *iPTH* intact parathyroid hormone, *RBC* red blood cells, *SGOT* serum glutamic oxaloacetic transaminase, *SGPT* serum glutamic pyruvic transaminase, *TSH* thyroid-stimulating hormone, *WBC* white blood cells

A longitudinal incision was made just lateral to the anterior tibial crest centered over the fracture site. The fascia over the tibialis anterior was divided, the muscle lifted off and the fracture site was visualized. The necrotic bone and callus at the fracture site was debrided with the use of an osteotome and a curette. Transverse drilling around the fracture site was done to promote healing and osteoblastic activity. Bone marrow from the ipsilateral iliac crest was inserted into the fracture site and a tension band plate was applied over the im nail.

**Fig. 2:**
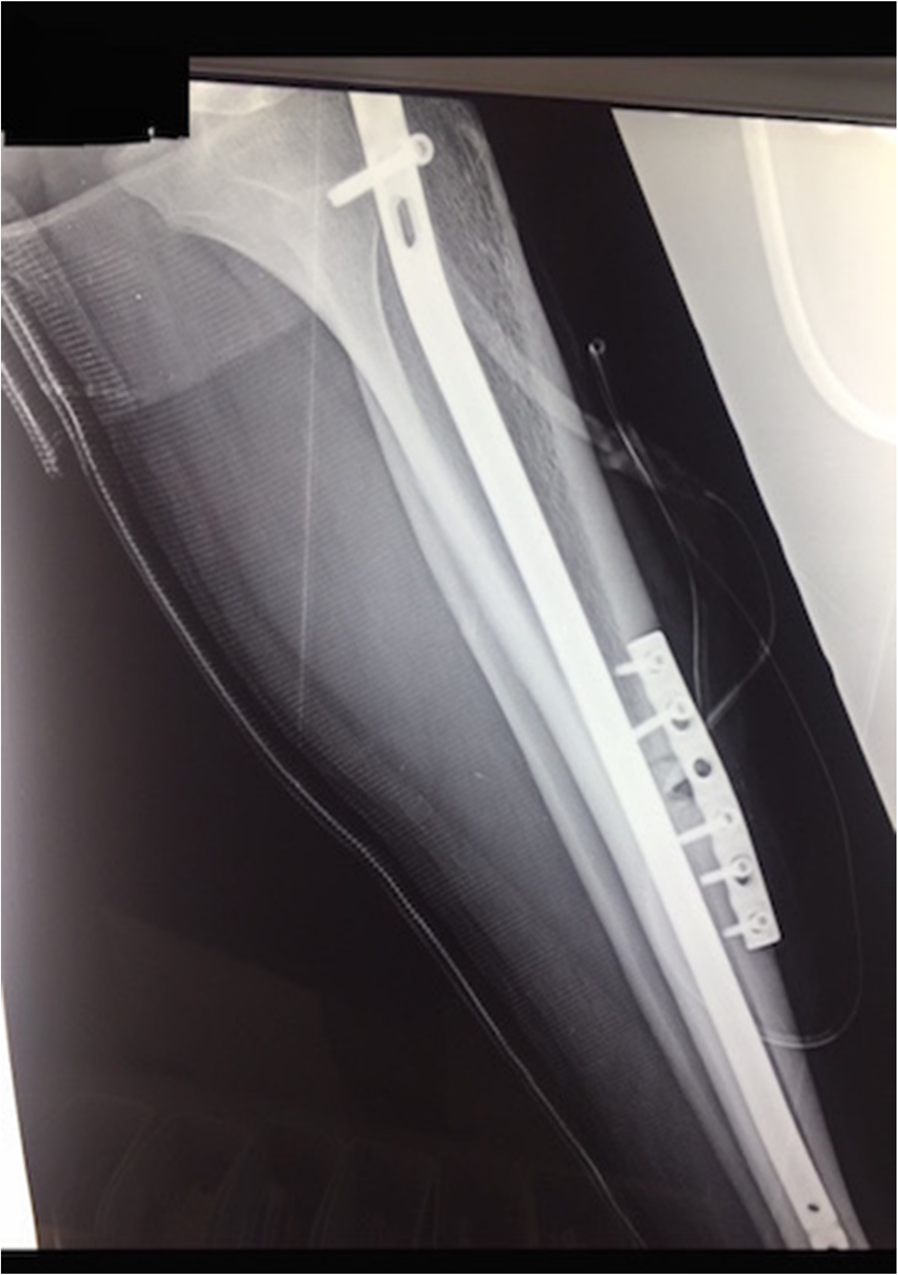
Tension band plating of the anterior tibia stress fracture nonunion. Immediately postoperative lateral view

We used a 6-hole, 4.5 mm locking compression plate. The plate was prebended and the screws were placed in a compression manner to achieve a tension band effect to the fracture site. A cortical screw was put first to the distal hole closest to the fracture site and then a cortical screw to the closest hole proximal to the fracture site to ensure compression of the fracture. Consequently, one unicortical locking screw was inserted proximally to the fracture site and the other two distally. With the use of locking and non-locking screws we minimized the pressure at the periosteum, which can damage blood supply to the poorly vascularized bone. The screws were angled in a different axis in order to bypass the nail (Fig. 2).

**Fig. 3:**
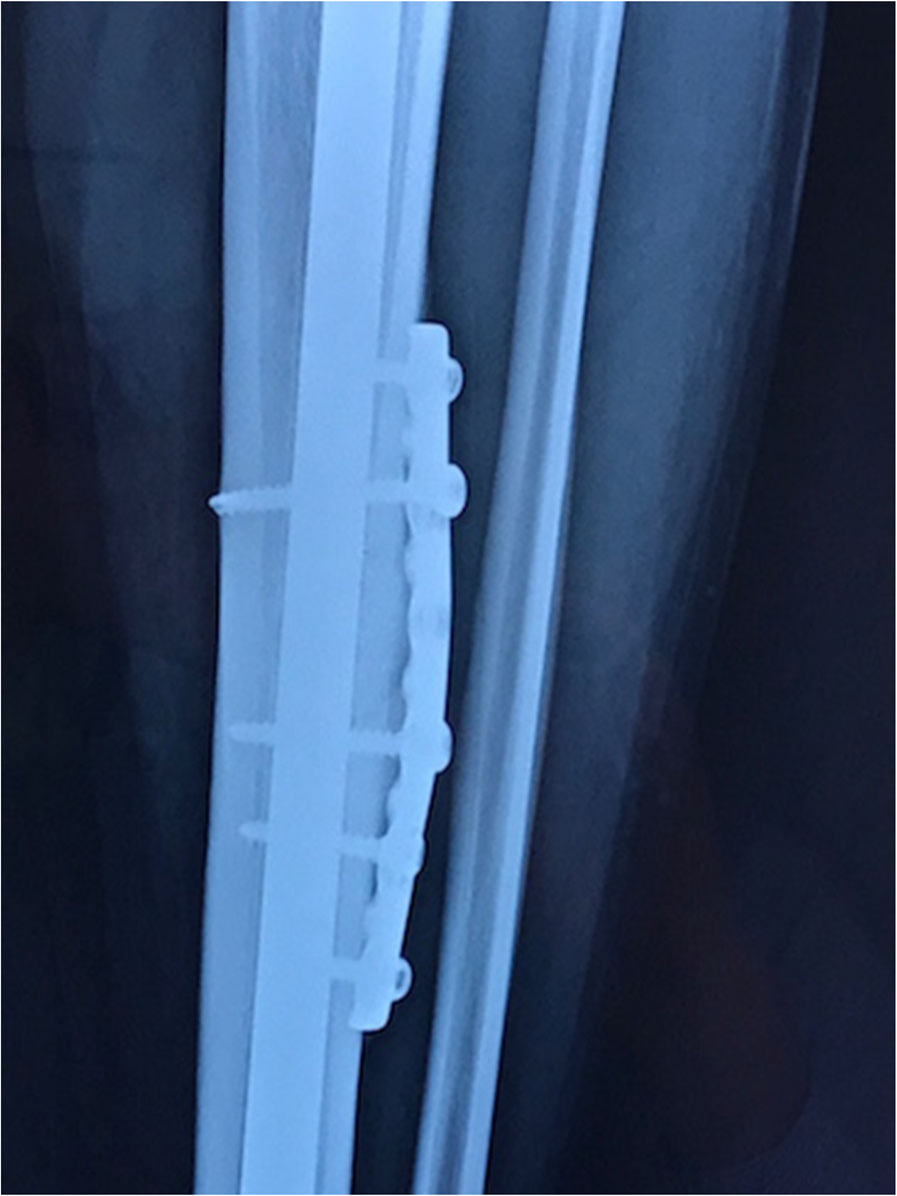
Tension band plating of the anterior tibia stress fracture nonunion. The union of the fracture is evident in this 6-month postoperative anteroposterior view

**Fig. 4:**
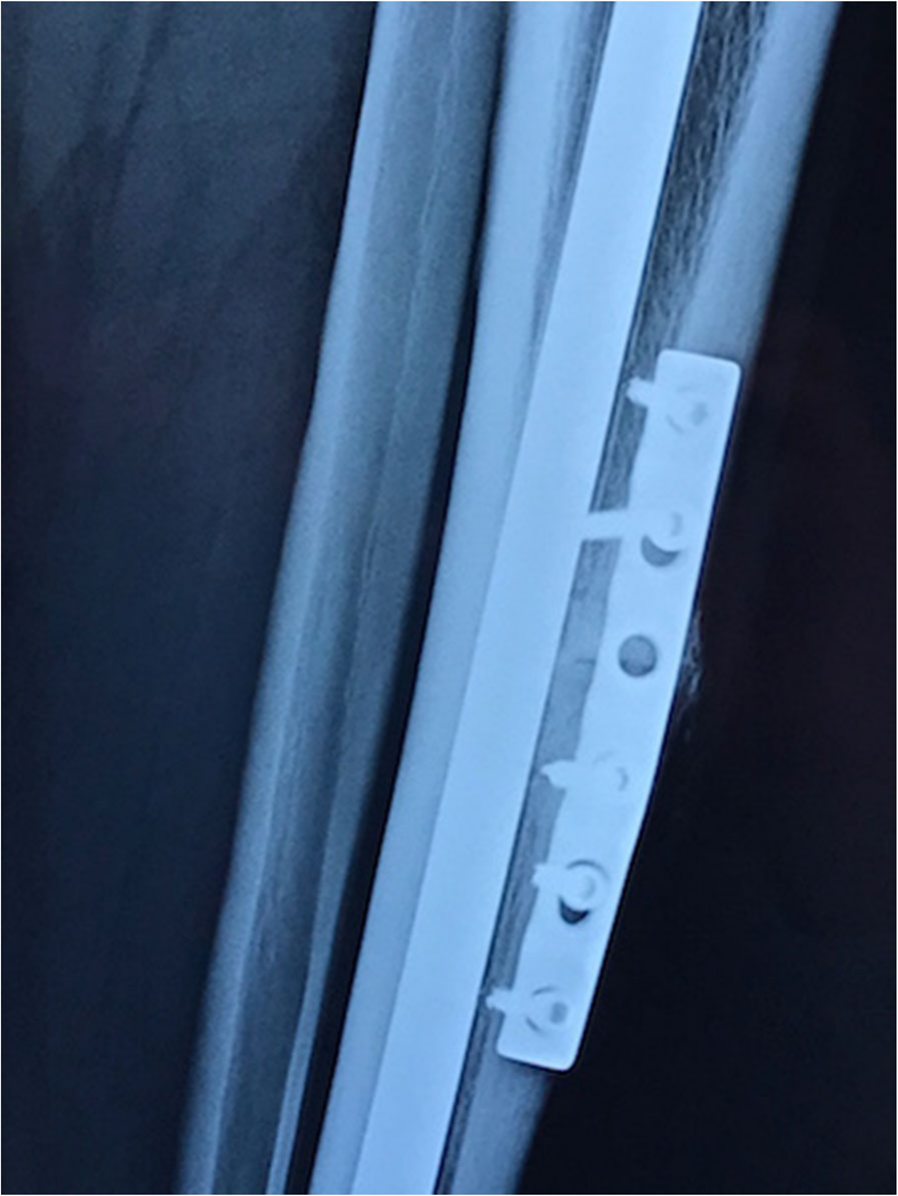
Tension band plating of the anterior tibia stress fracture nonunion. The union of the fracture is evident in this 6-month postoperative lateral view

Postoperatively, our patient was advised to wear an orthotic boot and to not bear weight for 6 weeks. Range of motion exercise involving knee and ankle and isometric exercises were initiated immediately postoperatively. After 6 weeks he progressed to weight bearing as tolerated. At 3 months postoperatively he was pain free and started light jogging, swimming, and plyometric and core stabilization exercises. At 6 months postoperatively the complete radiologic union of the fracture was evident (Figs. 3 and 4). He was symptom free; he resumed at that time a full training program and he returned to play football 6 months postoperatively at his preinjury high competition level.

## Discussion

We presented a case of a young elite football player who suffered from an anterior tibial stress fracture. Initially, he was treated conservatively. When it became obvious that the fracture would not heal this way, im nailing was performed. Unfortunately, the fracture did not heal again, so we decided to perform tension band plating without removing the im nail. With this technique, union at the fracture site was achieved and the athlete returned to play football, 6-months postoperatively. To the best of our knowledge, this is the first reported case in which tension band plating was placed in order to treat an anterior tibial stress fracture nonunion, without removing the preexisting im nail.

With this case report we try to consider two things:

First, among other treatment options such as im nail exchange and bone grafting, tension band plating over the nail is a worthwhile and valuable option to treat non-united tibial stress fractures treated previously with im nailing.

Second, we considered what the optimal surgical treatment for the special category of anterior tibial cortex stress fractures would be. For the reasons discussed below, tension band plating may be superior to im nailing, not only when nailing fails, but also as a first-line surgical treatment of anterior tibial cortex fracture when conservative treatment fails and surgery is indicated. We hypothesize that im nailing may be preferable for posteromedial tibial stress fractures, but tension band plating is preferable for anterior tibial cortex stress fractures.

Anterior tibial stress fractures in athletes are a challenge for clinicians. Non-operative treatment, such as rest, braces, ultrasound therapy, or electromagnetic field therapy, is associated with prolonged healing time and delayed time to return to play, which also has psychological effects on athletes. Moreover, delayed union, nonunion, and progression to complete fracture is common with conservative treatment [12].

Non-operative treatment is, in general, the primary treatment for stress fractures; however, for anterior tibial stress fractures it may not be the optimal treatment. These fractures are resistant to non-operative healing due to high tensile load, poor local vascularity, and lack of adequate muscle and soft tissue coverage, which all compromise bone healing. For this reason, early surgical intervention should be considered for anterior tibial stress fractures [13].

We believe that im nailing is an optimal treatment for posteromedial tibial stress fractures, but tension band plate is favorable for anterior tibial stress fractures. In addition, insertion of the tibial nail violates the knee joint, disrupts the extensor mechanism, and can cause anterior knee pain with kneeling and bending. The use of tension band plating is an effective treatment without the potential risks of im nail insertion [14, 15].

Tension band plating offers biomechanical advantages over im nailing [16, 17]. It provides compression at the tension side of the tibial cortex and counteracts tensile forces from the posterior muscle group. The distance of the plate from the central axis of the bone is another factor that alleviates tensile forces, fracture opening displacement, and motion. The tension band plating technique enables the surgeon to debride nonviable tissue, perform local excision of necrotic bone in cases of nonunion of tibial stress fractures, as in our case, and drilling and grafting at the fracture site. All these may be possible with im nailing too, but require an additional incision over the fracture site.

## Conclusions

We presented a case of tension band plating of an anterior tibial stress fracture nonunion, treated previously with im nailing. The plate was placed over the nail and the fracture healed properly. We believe that anterior tension band plating is superior to im nailing in managing anterior tibial stress fractures. It is a reliable option not only after failure of im nailing, but also as a first-line surgical treatment of anterior tibial cortex stress fractures. This technique offers advantages, such as no violation of the extensor mechanism and risk of anterior knee pain, and directly addresses the underlying problem of distraction forces acting on the anterior tibial cortex compromising fracture healing. Especially in high-level athletes, who cannot tolerate prolonged inactivity, early surgical intervention of acute or even non-united anterior tibial stress fractures with tension band plating is a reliable option that can accelerate recovery and return to play. Of course, it is a single case and further studies are needed.
